# High-Performance Microbial Fuel Cell for Aromatic Hydrocarbon Bioremediation: Leveraging a Unique Mangrove-Derived Electrogenic Consortium

**DOI:** 10.1021/acsomega.5c05703

**Published:** 2025-10-22

**Authors:** João Carlos de Souza, Ana Clara Bonizol Zani, João Pedro Silva, Amanda dos Santos, Gisela de Aragão Umbuzeiro, André Valente Bueno, Fernanda Leite Lobo, Valeria Reginatto, Adalgisa Rodrigues de Andrade

**Affiliations:** † 124588 University of São Paulo (USP), Faculty of Philosophy, Sciences and Letters at Ribeirão Preto (FFCLRP), Department of Chemistry, Avenida Bandeirantes 3900, Ribeirão Preto, São Paulo State 14040-900, Brazil; ‡ Universidade Estadual Paulista Julio de Mesquita Filho (UNESP), Instituto de Química, Departamento de Analítica, Rua Prof. Francisco Degni 55, Araraquara, São Paulo State 14800-060, Brazil; § State University of Campinas, Faculty of Technology, Rua Paschoal Marmo 1888, Limeira, São Paulo State 13484-332, Brazil; ∥ 28121 Federal University of Ceará, Technology Center, Department of Mechanical Engineering, Avenida da Universidade 2853, Fortaleza, Ceará State 60020-181, Brazil

## Abstract

Enhancing bioelectrocatalytic activity to increase the efficiency of toxic compound biodegradation and energy generation continues to be a critical challenge in bioelectrochemical systems. In this context, the present study aimed to obtain a novel electrogenic microbial consortium, sourced from mangrove sediments, capable of improving microbial fuel cell (MFC) performance in both energy generation and aromatic compound’s biodegradation. This new microbial consortium was tested in dual-chamber MFCs designed for the biodegradation of benzene, employed as a model aromatic compound. Overall, the results demonstrated that the enrichment of a microbial community derived from mangrove sediments in the southeastern region of Brazil (State of Espírito Santo) significantly enhanced bioelectricity generation in MFCs via benzene biodegradation. During the initial acclimation phase using 1000.0 mg L^–1^ sodium acetate, the bacterial genera *Arcobacter* (20.2%) and *Comamonas* (11.0%) were predominant. As sodium acetate was progressively replaced and the MFC operated solely with benzene (330.0 mg L^–1^), *Bacillus* (32.9%) and *Arcobacter* (30.3%) became the dominant genera. The MFC exhibited remarkable efficiency, achieving 98.7 ± 2.4% benzene removal within 96 h, while the output voltage increased from 568.0 ± 10.3 mV to 902.3 ± 20.6 mV as the feedstock shifted from sodium acetate to benzene. The maximum power density, Coulombic efficiency, and MFC cumulative energy efficiency were 390.1 ± 26 mW m^–2^, 14.4%, and 17.8%, respectively, surpassing previously established benchmarks and improving power density by approximately 100-fold compared to other devices. In conclusion, this innovative electrogenic microbial consortium, characterized by its unique bacterial diversity, markedly enhanced electron transfer, voltage, power density, and current generation in MFCs. It represents a highly promising and sophisticated approach for both substantial bioelectricity production and the effective bioremediation of aromatics, especially benzene, a compound known for its extreme toxicity, mutagenicity, and carcinogenicity.

## Graphical abstract

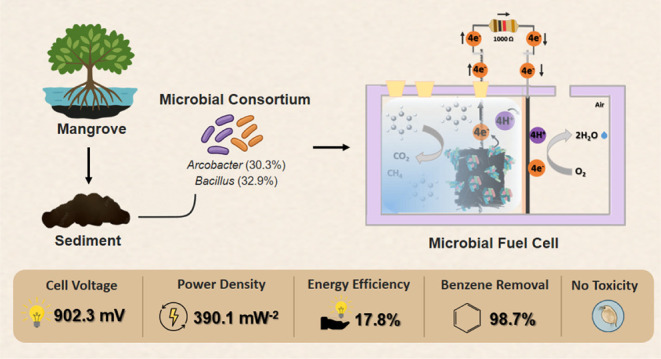

## 1 Introduction

Rapid population growth, intense urbanization, and the expansion of industrialization, combined with an economy and lifestyle based on consumption, have raised concerns among leaders, researchers, and experts from various fields of knowledge around the world regarding the adequacy of the current energy supply to sustain the production chain and related activities.^1^

Currently, the primary sources of energy in use largely depend on the combustion of fossil fuels, such as oil, coal, and natural gas.^2^ However, both the extraction and combustion of these fuels result in significant harm to public health and the environment as a whole.^2–5^ Particularly noteworthy is the rising incidence of soil, water, and air contamination caused by petroleum and its derivatives, such as benzene, which are often linked to oil extraction, refining, spills, improper disposal of petroleum industry waste, and emissions from engine combustion.^3,5–7^

Benzene is a highly genotoxic, mutagenic, carcinogenic, and toxic compound that presents significant challenges for treatment and removal.^3,8–12^ Furthermore, it contributes to environmental degradation by depleting the ozone layer, exacerbating the greenhouse effect, and promoting acid rain formation.^3,5^

In this context, bioelectrochemical devices, such as microbial fuel cells (MFCs), have garnered considerable attention as a promising alternative for clean energy generation, alongside the simultaneous bioremediation and biodegradation of compounds that pose serious risks to human health and the environment. This is because various types of waste or contaminants can serve as substrates or fuel sources in MFCs.^13–17^ In summary, MFCs are bioelectrochemical systems capable of converting the chemical energy of pollutants into electrical energy through their oxidation, using microorganisms as biocatalysts.^18^

Nonetheless, the low power density generated by MFCs remains a significant challenge, hindering their large-scale implementation.^19^ To address this limitation, research efforts have focused on developing new MFC designs with varying sizes and materials, implementing continuous feeding strategies with one or multiple substrates, coupling MFCs in series or parallel configurations, employing diverse substances in the medium composition, and optimizing the structural configuration of cathodes and anodes to reduce internal resistance and enhance electron transfer. Additionally, advancements include the incorporation of suitable microbial consortia to improve MFC performance.^14,19–27^

Recently, several microbial species have been isolated, and their ability to degrade highly toxic compounds, such as benzene and its derivatives, in different matrices has been extensively investigated.^28^ *Pseudomonas* has been the most studied genus in this context,^29–31^ although other bacterial species, such as *Rhodococcus rhodochrous*,^32^ *Acinetobacter baumannii*,^33^ *Geobacter*,^34^ *Microbacterium esteraromaticum* SBS1-720,^35^ *Paraburkholderia aromaivorans*,^36^ and *Variovorax paradoxus* ^37^ have also been subjects of investigation.

Another crucial aspect that determines microbial fuel cell (MFC) performance is the delivery of electrons from microorganisms to the electrode, a process known as extracellular electron transfer (EET). *Shewanella* and *Geobacter* species, both Gram-negative bacteria, have served as model organisms for studying EET due to their distinct mechanisms. *Shewanella* species utilize outer membrane vesicles connected to outer membrane extensions. These electron-rich extensions facilitate electron delivery to an extracellular acceptor. In contrast, *Geobacter* species utilize conductive protein filaments as cellular extensions. These filaments, identified as conductive pili (or e-pili), are composed of micrometer-long chains of cytochromes.^38^

Although still not extensively explored, electron transfer in Gram-positive bacteria has been studied, and some research has demonstrated the exoelectrogenic potential of *Bacillus* species in MFCs.^39–41^ For instance, electron transfer in *Bacillus cereus* is achieved through a dual mechanism. One method involves direct contact, requiring the alignment of the cytochrome complex, while the other is indirect, involving the release of flavin molecules into the solution, which act as mediators.^42^ In addition, the genus *Bacillus* has also been identified as capable of degrading poorly degradable molecules.^43^ In this context, Duarte-Urbina et al.^39^ developed a bioanode using biochar-based catalysts derived from onion waste, combined with a *Bacillus subtilis* strain, for the treatment of pharmaceutical waste in MFCs. The study showed the formation of a stable biofilm with good electron transfer capacity, achieving a power density of 30.72 mW m^–2^ and a 42.4% reduction in chemical oxygen demand after 14 days of operation. Guo et al.^40^ evaluated the performance of *Bacillus cereus* in oily sludge treatment and energy generation. The results demonstrated that the bacterium alone achieved 87.76% oil removal and a maximum power density of 65 mW m^–^². Wongbunmak et al.^28^ investigated both the capacity and degradation pathway of benzene and its derivatives using the *Bacillus amyloliquefaciens* subsp. plantarum W1 strain in a nonbioelectrochemical system. Their results demonstrated approximately 50% degradation of benzene and its derivatives after 7 days.

Furthermore, given that EET can occur in both redox directions, microorganisms are capable of direct cell-to-cell electron transfer, where one bacterium acts as the electron acceptor and another as the electron donor. Therefore, the discovery of new microbial consortia is of paramount importance, as it is the electrogenic microorganisms that transfer electrons from degraded compounds through oxidation, thereby enabling the generation of electricity in MFCs.^44–46^ Consequently, the present study proposes a highly promising and viable approach to enhance both bioelectricity generation and benzene (as a model) biodegradation in MFCs through the tailoring of a novel microbial consortium derived from mangrove sediments.

## 2 Materials and Methods

### 2.1 Reagents and Solutions

All the reagents were of analytical grade. The aqueous solutions were prepared by using ultrapure water from a Milli-Q System (Millipore). Benzene (C_6_H_6_, 99.0%) was obtained from Exôdo Científica. Potassium ferricyanide (K_3_Fe­(CN)_6_, 99.0%) and potassium ferrocyanide (K_4_Fe­(CN)_6_, 99.0%) were acquired from Qhemis. Hydrochloric acid (HCl, 36.5–38.0%) was purchased from Vetec. Potassium chloride (KCl, 99.5%), potassium bicarbonate (KHCO_3_, 99.5%), sodium hydrogen phosphate (Na_2_HPO_4_, 99.0%), sodium dihydrogen phosphate dihydrate (NaH_2_PO_4_.2H_2_O, 99.0%), ammonium chloride (NH_4_Cl, 99.5%), magnesium sulfate (MgSO_4_, 99.5%), manganese chloride tetrahydrate (MnCl_2_.4H_2_O), sodium molybdate dihydrate (NaMoO_4_.2H_2_O, 99.5%), and dimethyl sulfoxide (DMSO, 99.5%) were supplied by Sigma-Aldrich. Ethanol (C_2_H_6_O, 100.0%), nitric acid (HNO_3_, 65.0%), hydrogen peroxide (H_2_O_2_, 100.0 volumes), sulfuric acid (H_2_SO_4_, 95.0–97.0%), sodium acetate (C_2_H_3_NaO_2_, 99.0%), sodium chloride (NaCl, 99.5%), and sodium hydroxide (NaOH, 98.0%) were obtained from Merck. Magnesium chloride (MgCl_2_, 99.0–102.0%) and calcium chloride dihydrate (CaCl_2_.2H_2_O, 99.0–105.0%) were acquired from Synth. Sodium bicarbonate (NaHCO_3_, 100.2%) was purchased from J. T. Baker. The yeast extract was obtained from Kasvi.

The Lovley and Phillips culture medium used in the experiments was prepared by employing the following mixture (g L^–1^): NaHCO_3_ (2.5), Na_2_HPO_4_ (0.74), NaH_2_PO_4_.2H_2_O (0.6), NH_4_Cl (1.5), MgCl_2_ (0.1), MgSO_4_ (0.1), yeast extract (0.05), CaCl_2_.2H_2_O (0.1), KCl (0.1), NaCl (0.1), NaMoO_4_.2H_2_O (0.001), and MnCl_2_.4H_2_O (0.005).^16^

### 2.2 MFC Structure

To carry out the experiments, six two-compartment MFCs (8.6 cm × 6.5 cm × 6.5 cm) were constructed by using acrylic (Figure 1).

**Figure 1:**
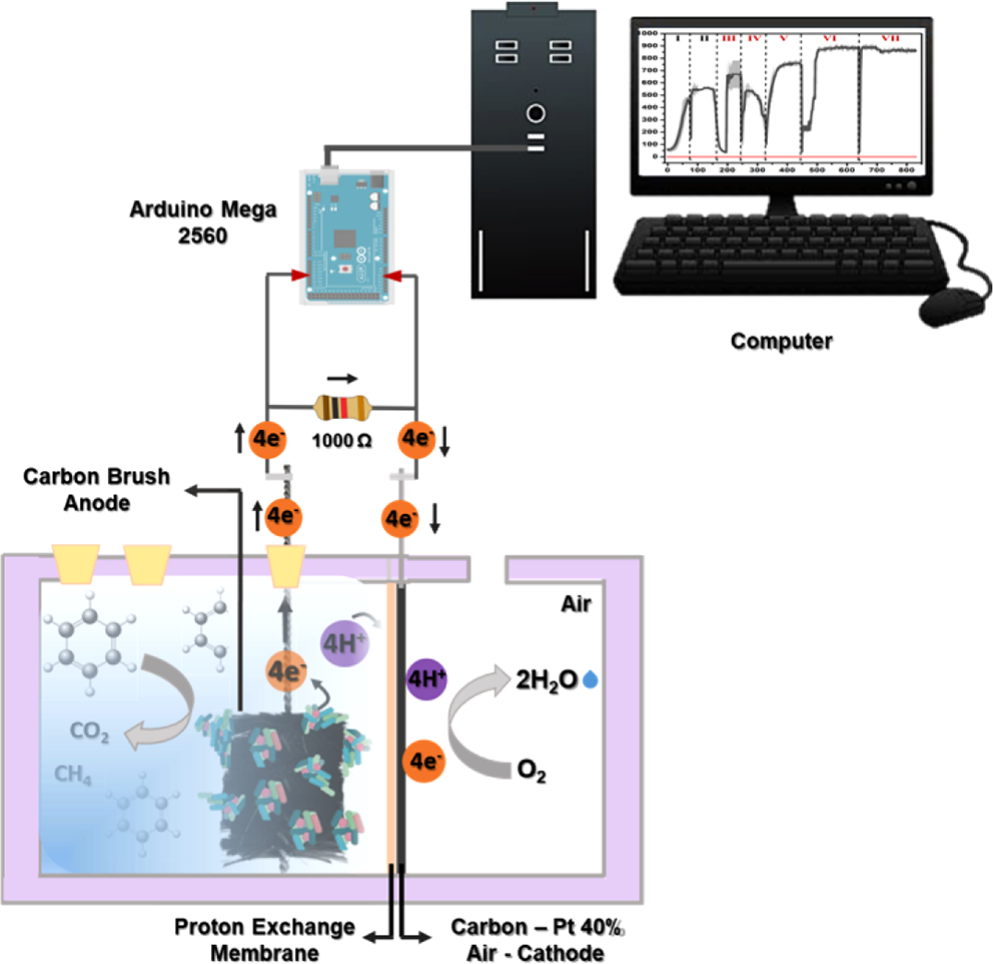
Schematic representation of the MFCs.

The anode compartment (4.8 cm × 6.5 cm × 6.5 cm), with a volumetric capacity of 50 mL, was equipped with an anode consisting of a carbon fiber brush (3.0 cm × 2.0 cm) connected to a titanium rod (Research Laboratory Shop, China), which was linked to the cathode through an external resistor of 1000 Ω, positioned at a distance of 0.5 cm. The cathode compartment (3.8 cm × 6.5 cm × 6.5 cm) contained perforations allowing contact with the ambient atmosphere. The cathodes were composed of a carbon cloth (15 cm^2^) modified with 40% platinum (A6 ELAT-BASF), which was hot-pressed at 130 °C and under 35 kgf cm^2^ onto a treated Nafion proton exchange membrane (NRE-212/Aldrich).

Prior to assembly and use, both the MFC units and the anode and cathode components were immersed for 24 h in a 20.0% (v/v) HNO_3_ solution, followed by thorough rinsing with ultrapure water. Specifically, the anodes were subjected to an additional heat treatment in a muffle furnace at 450 °C for 45 min after rinsing.

The voltage generated by the MFCs was recorded in real time every 15 min using an Arduino Mega 2560 microcontroller board (ATmega2560) connected to a microcomputer.

### 2.3 Biofilm Growth on the Anode and MFC Functioning

A mangrove sediment originating from Vitória, Espírito Santo State, Brazil (Latitude: 20°16′45.3′’S; Longitude: 40°18′27.4’’W) was used as inoculum in the MFCs. For this purpose, 50.0 mL of a 20.0% (m/v) suspension composed of mangrove sediment and Lovley and Phillips medium was transferred to the anodic compartment.

Initially, a concentration of 1000.0 mg L^–1^ of sodium acetate (SA) was used to feed the microorganisms over a period of 7 days.^16^ On the third day, an additional supply of SA was introduced. After 8 days, the original solution and sediments in the anode compartment were removed, and a fresh solution of the culture medium, along with SA, was added.

To establish a stable biofilm, two consecutive feeding cycles with sodium acetate (SA) were conducted at intervals of 3 to 5 days, initiated whenever a decrease in output voltage was observed. Subsequently, SA was gradually replaced by benzene at concentrations representing 10.0%, 25.0%, 50.0%, 75.0%, and 100.0% of the total carbon content, corresponding to benzene concentrations of 30.0, 80.0, 170.0, 250.0, and 330.0 mg L^–1^, respectively. As a control, a microbial fuel cell (MFC) without added sediment (abiotic condition), containing only the culture medium and the carbon source, was used. All experiments were carried out at a controlled room temperature (25 ± 2 °C).

### 2.4 MFC Electrochemical Characterization

The electrochemical measurements were carried out using an AUTOLAB potentiostat/galvanostat, model PGSTAT 30 (Metrohm AUTOLAB, Switzerland), connected to a microcomputer. To obtain and interpret the data, the NOVA 2.10 software was used.

Cyclic voltammetry measurements were conducted at a scan rate (ν) of 1 mV s^–1^ within the MFC itself, using the Lovley and Phillips medium as the supporting electrolyte. These measurements were carried out in the presence of sodium acetate (SA), benzene, or under abiotic conditions. An Ag/AgCl_(sat)_ electrode (3.0 mol L^–1^ KCl) served as the reference electrode

The power density tests of the MFC were determined under potentiostatic conditions using linear sweep voltammetry at ν = 1 mV s^–1^. Initially, the MFCs were maintained at open circuit potential (OCP) for 3 h or until the voltage stabilized. Measurements were then conducted by sweeping the potential from OCP to zero voltage. Power density was calculated using eq 1:^16^

1 $$P=\frac{iU}{A}$$

where *P* is the power density (W m^–2^), *i* is the current (A), *U* is the cell voltage (V), and *A* is the anode geometric area (m^2^).

The internal resistance of each MFC was calculated by using eq 2:^47^

2 $$Rint=\frac{U}{i}-Rext$$

where *Rint* is the internal resistance (Ω) and *Rext* is the external resistance (Ω).

Chronoamperometric measurements were conducted in the MFC at a fixed potential of 0.2 V vs Ag/AgCl_(sat)_ (3.0 mol L^–1^ KCl) for a duration of 3600 s, using the Lovley and Phillips medium as the supporting electrolyte. These measurements were performed in the presence of either sodium acetate (SA) or benzene.

The coulombic efficiency (CE) of the benzene-fed MFC was calculated by using eq 3:^16^

3 $$CE=\frac{MMs\times I\times t}{F\times b\times V\times \Delta C}$$

where *MMs* is the substrate molar mass (benzene = 78.11 g mol^–1^), *I* is the current (A), *t* is the time (s), *F* is the Faraday constant (96485 C mol^–1^), *b* is the total number of electrons produced for substrate oxidation (*n* = 30 for benzene total oxidation), *V* is the MFC anodic compartment volume (L), and *Δ*C is the substrate concentration (g L^–1^).

Electrochemical impedance spectroscopy (EIS) measurements were carried out at OCP under the same conditions described previously. The frequency was varied from 60 kHz to 0.01 Hz, with ten frequency values per decade, using a root-mean-square sinusoidal disturbance (r.m.s.) with an amplitude of 10 mV.

The electrochemical surface area (ECSA) of the carbon fiber brush in the absence and presence of biofilm was calculated by using eq 4:^16^

4 $$ECSA=\frac{Cdl}{Cs}$$

where *C_dl_* is the double layer capacitance and *C_s_* is the graphite-specific capacitance (0.0043 mF cm^–2^).^16^

### 2.5 Anode Surface Morphological Characterization

The surface of the anode was characterized using scanning electron microscopy (SEM) both before and after biofilm formation and acclimatization in the presence of SA or benzene. A Carl Zeiss scanning electron microscope, model EVO 50 (Cambridge, United Kingdom), in high vacuum mode (10^–5^ Torr), with an electron beam acceleration voltage of 20 kV and equipped with a secondary electron detector, was used. For this purpose, carbon fiber brush filaments were immersed in an Eppendorf flask containing 2.0% (m/v) glutaraldehyde at 4 °C for 3 h. Then, the filaments were treated with 1.0% (m/v) osmium tetroxide for 2 h. To preserve the microorganism shape, the biofilm present on the filaments was dehydrated by employing different water/ethanol ratios and subsequently dried to the critical point. Finally, the dried filaments were coated with gold by the sputtering technique (Bal-tec, SCD 050, Fürstenstein, Liechtenstein).

Subsequently, bacterial viability before and after biofilm formation with SA and benzene was assessed by confocal laser scanning microscopy (CLSM) analysis using a LIVE/DEAD Viability/Cytotoxicity Kit (Invitrogen, Thermo Fisher Scientific). For CLSM analyses, an AxioObserver inverted microscope, model LSM 780, from Carl Zeiss (Jena, Germany), was used. The viability kit contains calcein-AM, which presents a green fluorescence when staining live bacterial cells. In contrast, the bacterial viability kit also contains ethidium homodimer-1, which presents red fluorescence when staining dead bacterial cells.^48^

### 2.6 Bioanode Microbial Diversity Analysis

Samples of the inoculum, the biofilm formed in the SA, and benzene were evaluated for microbial diversity.

The bioanodes formed in the MFC were vortexed for two 5 min intervals in sterile 0.9% (m/v) NaCl to obtain the cells that remained attached to the anode.

Metagenomic DNA was extracted from centrifuged anodic biofilm pellets by using the ZymoBIOMICS^TM^ DNA Miniprep Kit (Zymo Research). The DNA samples were quantified and analyzed for quality and quantity. Then, the DNA samples were subjected to amplification of the entire 16S rRNA gene by employing primers 27F and 1492R (∼1.6 kb fragment). The amplicons were visualized on agarose gel and quantified. The resulting fragments were used to construct sequencing libraries and were sequenced on the MinION platform (Oxford Nanopore) by employing Flongle Flow Cells (FLO-FLG001).

The resulting reads, obtained after sequencing, were subjected to base calling by using the Guppy Basecaller GPU version (v.6.0.7). Furthermore, the reads were filtered for quality control in Q10 by employing NanoFilt (v.2.3.0), demultiplexed with Porechop (v.0.2.4), and mapped to the 16S reference database by using the KMA tool (v.1.4.3). The data were analyzed by employing Python 3.7.

### 2.7 Monitoring Benzene Biodegradation and Products

Benzene biodegradation in the MFC was monitored by gas chromatography coupled to a flame ionization detector (GC-FID). For this, an analytical methodology adapted from Souza et al.^3^ was used. For the analysis, a Shimadzu chromatograph model GC-2010 Plus, Restek Rtx – Biodiesel TG chromatographic column (5% diphenyl–95% dimethyl polysiloxane cross-linked; 10 m × 0.32 mm i.d., 0.10 μm d.f.), and nitrogen (purity 99.995%) at a flow rate of 1.0 mL min^–1^ as a carrier gas^,^ were employed. The column and injector temperatures were kept constant at 40.0 and 240.0 °C, respectively. The injector was operated in split mode (1:50).

Benzene was detected and quantified by using the headspace technique in an automatic headspace injector (CTC Analytics – Combi Pal). For this purpose, 2.0 mL of the sample was transferred to a closed bottle (10 mL). Subsequently, the bottle was heated under stirring at 65.0 °C for 5 min. Then, the volatile fraction was injected into the GC and analyzed.

Monitoring of the products in solution was carried out by high-performance liquid chromatography (HPLC), using a Shimadzu HPLC system, model LC-20AT, coupled to a refractive index detector (RID). To conduct HPLC-RID analysis, MFC medium samples were collected in different periods, filtered through 0.22 μm syringe filters, and analyzed directly. The separation was performed on an Aminex HPX-87H ion exclusion column (300 × 7.8 mm), combined with a cation H column guard (30 × 4.6 mm). The analysis was carried out in isocratic mode with a mobile phase composed of 5.0 mmol L^–1^ H_2_SO_4_, flow rate of 0.6 mL min^–1^, a column temperature of 60 °C, and an injection volume of 10.0 μL.

Monitoring of the products in the gaseous fraction was carried out by gas chromatography coupled to a thermal conductivity detector (GC-TCD), using a Shimadzu gas chromatograph, model GC-2014. To carry out the analyses, a volume of 50.0 μL of the MFC gas fraction was collected with a GC injection syringe and injected directly into the gas chromatograph. Separation was performed on a Restek Shin Carbon ST – Micropacked chromatographic column (SilcoSmooth Tubing Mesh: 100/120 OD: 1/16”; 2 m length × 1.0 mm i.d.). Analysis was performed in splitless mode with the compound carrier gas argon (99.9995%), a flow rate of 10.0 mL min^–1^, a column temperature of 50.0 °C, and a detector temperature of 80.0 °C. LabSolutions software was also used for data acquisition and processing.

All measurements were performed in triplicate, and quantifications were carried out using analytical curves developed for each identified product.

### 2.8 Acute Toxicity Tests

Initially, the pure Lovley and Phillips medium was diluted in synthetic culture medium for *Daphnia similis* ^49^ at concentrations of 0.1, 0.3, 1.0, 3.3, and 10.0% (v/v) to test for toxicity.

Subsequently, the toxicity of the Lovley and Phillips medium in the presence of benzene at 0.33, 1.1, 3.3, 11.0, and 33.0 mg L^–1^ and after benzene degradation in the MFC was evaluated. To this end, both media were diluted in synthetic medium for *D. similis* at 3.3% (v/v) in the presence of 0.01% (v/v) DMSO.^49–51^ Synthetic medium^49^ was used as the negative control, and 0.01% (v/v) DMSO in synthetic medium was used as blank.

*D. similis* cultivation and acute toxicity tests were carried out according to ABNT NBR 12713:2022 ^52^ and OECD guidelines 202.^51^ NaCl was used as a reference to monitor culture sensitivity, and only cultures within the acceptable range were employed in the test. The neonates (*n* = 20, ≤24 h) were obtained from two- to three-week-old mothers and transferred to four replicates for each concentration. The tests were performed at 21 ± 1 °C, under a 16-h light and 8-h dark cycle, and the number of immobile organisms was recorded after 48 h. Mortality above 10% indicates toxicity.

## 3 Results and Discussion

### 3.1 MFC Performance

Biofilm formation, development, and stability were evaluated by monitoring the output voltage of the MFC during its operation. Figure 2 presents the voltage profiles generated by both the control and biotic MFCs.

**Figure 2:**
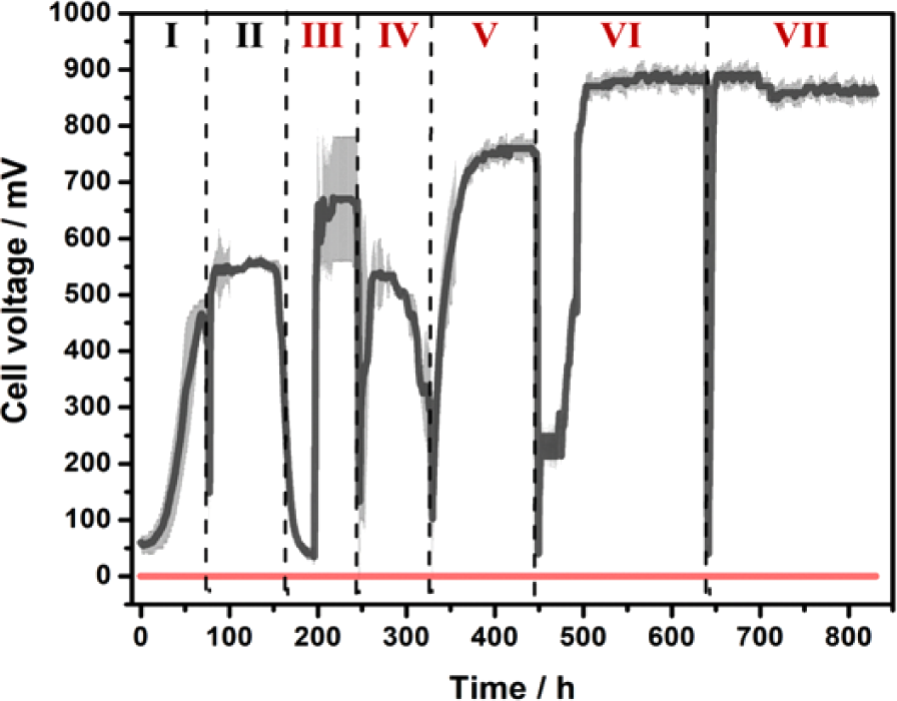
Voltage measurements of the abiotic control (**—**) and MFCs (**—**) during feed cycles with 1000.0 mg L^–1^ SA (**I** and **II**) and benzene at 30.0 (**III**), 80.0 (**IV**), 170.0 (**V**), 250.0 (**VI**), and 330.0 mg L^–1^ (**VII**) in Lovley and Phillips medium. Conditions: *R*ext = 1000 Ω.

Cycles I and II (Figure 2), conducted with 1000.0 mg L^–1^ of sodium acetate (SA), exhibited maximum voltages of 470.5 ± 25.1 mV and 568.0 ± 10.3 mV, respectively. Starting from Cycle III, SA was gradually replaced by benzene, facilitating the acclimatization of the biofilm and preserving the MFC’s energy generation efficiency.^16^ This strategy mitigates the effects of molecular recalcitrance and reduces the acute toxicity of benzene to microorganisms. Maximum voltages observed in Cycles III, IV, V, VI, and VII were 678.5 ± 98.2, 542.4 ± 50.7, 768.6 ± 10.8, 902.3 ± 20.6, and 899.9 ± 15.2 mV, respectively.

The voltage outputs recorded for the MFCs indicated the development of an electroactive biofilm; also, the abiotic MFC control does not signalize any voltage (Figure 2). Biofilm formation follows a cyclical pattern involving interactions between microorganisms and the anode surface,^53–55^ which corresponds to the voltage increase observed in Cycle II (21.0% higher than Cycle I). Remarkably, the introduction of benzene (30.0 mg L^–1^) in Cycle III led to a further voltage increase compared to the previous cycles, indicating a stable biofilm. Although the benzene concentration was still low (30.0 mg L^–1^), the biofilm demonstrated a promising level of adaptability to the new substrate.

In Cycle IV, the voltage decreased slightly, probably due to the increased amount of benzene (80.0 mg L^–1^). This may have inhibited the electrogenic microorganisms, which were not yet fully adapted to the increased presence of benzene. After Cycle V, the voltage increased considerably (42.0% compared to Cycle IV), even with the addition of a higher concentration of benzene, indicating that the biofilm had adapted well to this toxic compound.

Biofilm regeneration was evaluated in the presence of SA (1000.0 mg L^–1^) and benzene (330.0 mg L^–1^) by cycling the MFC from external load (*R*ext = 1000 Ω) to short-circuit (*R*ext = 0) every 24 h during a single-batch experiment. As shown in Figures S1 and S2 for SA and benzene, respectively, the voltage output stabilized 24 h after feeding. After removing the external resistance, there was an increase in voltage. Without external resistance, electron flow is maximized at the anode, increasing the potential difference. At this point, electrogenic microorganisms compete for the substrate equally with other suspended microorganisms present in the MFC.^16^ However, when reconnecting the external resistance, the activity of electrogenic bacteria adhered to the anode surface is privileged.^16^ This demonstrates that the microorganisms are able to self-regulate accordingly to produce energy. Furthermore, they were adapted to the presence of benzene as a new carbon source, forming a very conductive, electroactive, and stable biofilm with excellent reactivation and energy production.

### 3.2 MFC Electrochemical Characterization

Initially, cyclic voltammetry measurements at ν = 1 mV s^–1^ were performed in the MFC itself in order to evaluate the electrochemical behavior of the anode before and after biofilm formation. A Lovley and Phillips culture medium was used as a supporting electrolyte in the presence of SA (1000.0 mg L^–1^) or benzene (330.0 mg L^–1^). The voltammograms obtained are shown in Figure S3 (Supporting Information).

The voltammograms obtained in the absence of biofilm (abiotic condition) did not show anodic oxidation or cathodic reduction peaks. In the presence of biofilm (biotic condition), anodic and cathodic peaks emerged for both SA and benzene (Figure S3). In the presence of SA alone (Figure S3A), the oxidation and reduction peaks appeared around 0.19 V *vs.* Ag/AgCl_(sat)_ (I = 1.87 ± 0.31 mA) and −0.27 V *vs.* Ag/AgCl_(sat)_ (I = 1.29 ± 0.17 mA), respectively. In the presence of benzene (Figure S3B), higher currents emerged; the oxidation peak appeared at 0.16 V *vs.* Ag/AgCl_(sat)_ (I = 3.47 ± 0.54 mA) and the reduction peak around −0.38 V *vs.* Ag/AgCl_(sat)_ (I = 1.68 ± 0.23 mA). These results showed that a conductive and electrochemically active biofilm formed on the anode surface for both SA and benzene. Although these redox potentials are compatible with direct electron transfer (DET) due to membrane-bound redox proteins, as reported previously,^56^ considering that the biofilm is formed by microbial consortia, where electron transfer often involves complex and cooperative interactions between different species, we cannot rule out that bacteria and the electrode may also exchange electrons by mediated electron transfer (MET).^15,56,57^

During DET, the cell membrane and electrode surface are in direct contact because electron transport occurs mainly via redox proteins (c-type cytochromes) or the electroconductive pili (type IV pili, also known as e-pili) of proteins present in these microorganisms (PilA protein).^58–61^ On the other hand, during MET, electron transfer is assisted by a mediator molecule that is generally excreted by the microorganisms.^58,62^ In this process, microorganisms transfer the electrons generated during their metabolism to a mediator molecule, which becomes reduced. The reduced mediator then migrates to the surface of the anode, where it donates the electrons to the external circuit and is reoxidized in the process. It subsequently returns to the microbial cell, where it is reduced again, thus restarting the electron transfer cycle.^58,62^

Well-characterized model organisms, such as *Geobacter sulfurreducens* and *Shewanella oneidensis* MR-1, provided insights into these mechanisms. *G. sulfurreducens* performs direct electron transfer (DET) via multiheme cytochromes C and type IV pili (e-pili) composed of the PilA protein.^44,63,64^ However, the mechanism is still debated. Some studies propose that stacked π orbitals of aromatic residues in PilA facilitate conduction, while others suggest that micrometer-long filaments of polymerized hexaheme cytochrome OmcS are responsible for long-range electron transport.^60,61^ In contrast, *S. oneidensis* employs both direct and mediated strategies. Its porin-cytochrome complex MtrCAB spans the outer membrane, enabling electron flow through a 20-heme relay and can also release soluble flavins to transport electrons to electrodes or other microbes.^44,65^

While these models offer more well-established mechanistic insights, microbial consortia present a more complex context for EET. In such communities, extracellular electron flow often results from cooperative and syntrophic interactions between diverse species, forming an interspecific electron transfer (IET).^66^ A notable example of extracellular EET was reported between *G. sulfurreducens* and *Clostridium pasteurianum*, in which *Geobacter* e-pili enabled direct electron transfer between species, avoiding the need for exogenous mediators and enhancing the potential for metabolic cooperation and complex electron networks within consortia, especially under diverse environmental or substrate conditions.^67^

When compared to well-established electroactive bacteria such as *G. sulfurreducens* and *S. oneidensis* MR-1, the mangrove-derived microbial consortium investigated here offers a distinct ecological and metabolic profile. While *Geobacter* and *Shewanella* have clearly defined EET pathways and typically require simple substrates such as acetate or lactate, the studied consortium thrives on complex carbon sources such as benzene. As shown in Section 3.3, the biofilm communities formed solely with benzene (330.0 mg L^–1^) exhibited a strong enrichment of *Bacillus* sp. The dominance of the genus *Bacillus* and other potential electroactive bacteria, such as *Arcobacter*, suggests an alternative EET, potentially involving DET and biofilm-based conduction. *Arcobacter* species are reported to perform EET through mechanisms that likely involve direct contact with external electron acceptors, facilitated by proteins such as multiheme c-type cytochromes, and potentially enhanced by structures like flagella that aid in biofilm formation and interaction with surfaces.^68^

SEM and CLSM images were obtained for the anode surface before and after biofilm formation (Figure 3).

**Figure 3:**
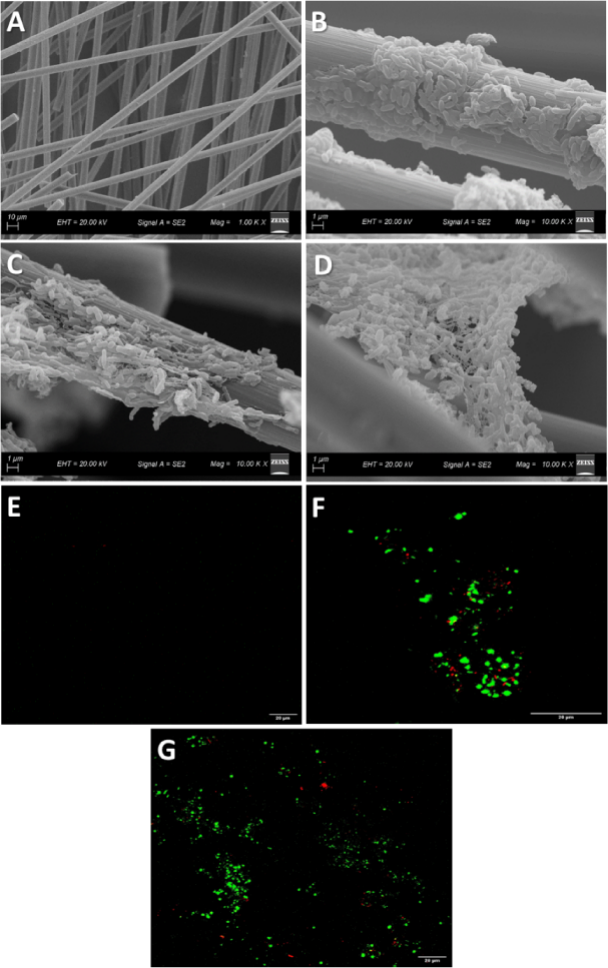
SEM images illustrating the anode surface prior to (A) and following biofilm formation in the presence of SA (B) and benzene (C,D), along with CLSM images of the anode surface before (E) and after biofilm formation in the presence of SA (F) and benzene (G).

Before biofilm formation, the carbon fiber brush used as an anode had bristles with a smooth and uniform surface (Figure 3A). After biofilm growth with SA, clusters of rod-shaped bacterial cells of different sizes were observed attached to the surface of the anode bristles (Figure 3B). However, in some regions, the clusters were interconnected by nanowire networks (Figure 3C,D), indicating that electron transfer may have occurred preferentially via DET on the anode surface. The ability of bacteria to transfer electrons via DET may favor the degradation of complex pollutant compounds, such as benzene. Therefore, electron transfer may have occurred via both DET and MET, since both can coexist in a mixed culture of microorganisms.^15,57^

Furthermore, bacterial viability before and after biofilm formation with SA and benzene can be observed (Figure 3E–G, respectively). Before biofilm formation (Figure 3E), there was no strong fluorescence, indicating low bacterial viability on the anode surface. However, after biofilm formation and growth with SA (Figure 3F), clusters of strong green fluorescence were observed with small spots of red fluorescence, indicating strong bacterial viability composed mainly of clusters of living organisms.

Finally, for the biofilm in the presence of benzene (Figure 3G), a greater spreading and greater bacterial viability were also observed, accompanied by a decrease in dead organisms compared to the biofilm in SA. Therefore, it can be inferred that a bacterial biofilm was formed and that bacterial activity is essentially established after the bacterial biofilm growth process. In addition, high biocompatibility of microorganisms can be observed both in the presence of SA and in the presence of benzene, since the biofilm formed is composed mainly of living organisms.

Figure 4 shows the polarization and power density (A) and chronoamperometric (B) curves for the MFC fed with SA (1000.0 mg L^–1^) or benzene (330.0 mg L^–1^). The polarization curve is generally divided into three regions. In the first region, located at lower current densities, polarization is activated, and the cell potential decreases. The second region, at moderate current densities, is closely related to the ohmic loss domain, but the cell potential drops more slowly and linearly. In the third region, at higher current densities, the cell potential drop loses linearity and is limited by pronounced concentration polarization.^69–71^

**Figure 4:**
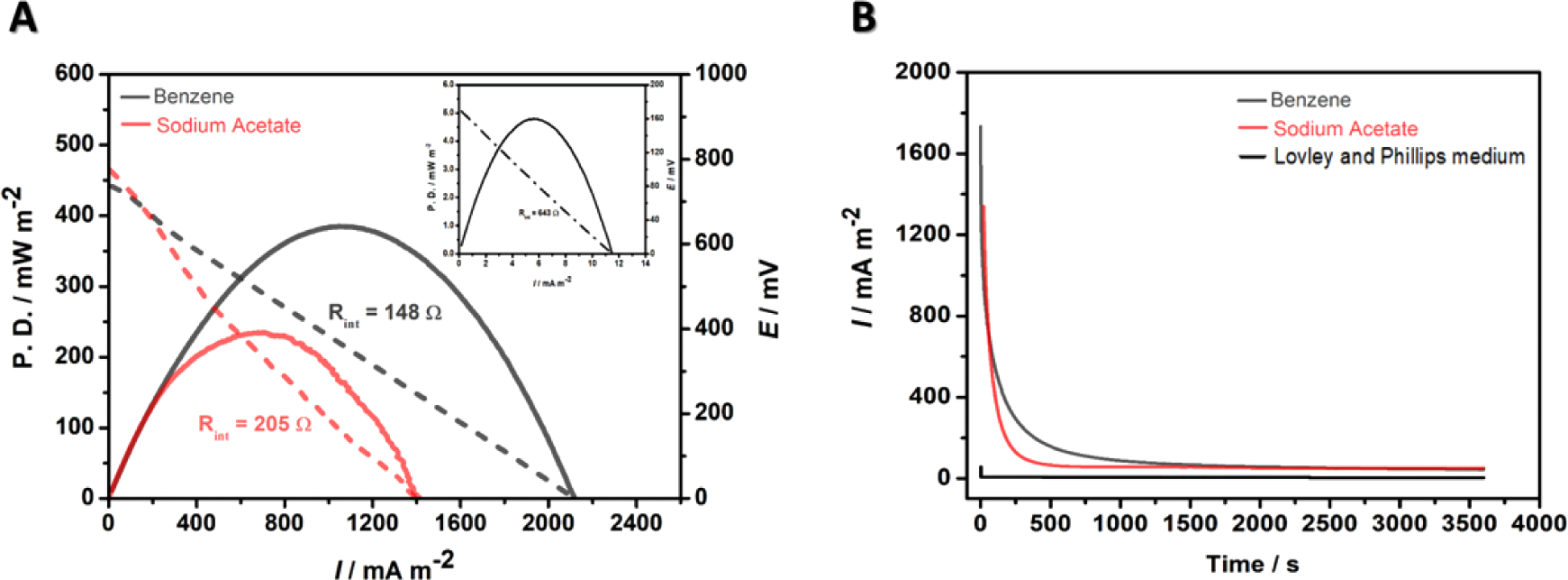
Polarization and power density (A) and chronoamperometric (B) curves of the MFC operating with the Lovley and Phillips medium (control - inset) and fed with 1000.0 mg L^–1^ SA (**—**) or benzene 330.0 mg L^–1^ (**—**).

The polarization and power density curves (Figure 4A) under abiotic and biotic conditions were symmetric, exhibited no hysteresis, and displayed only the first two overpotential regions. The maximum power density obtained from the polarization curves (Figure 4A) was 4.8 ± 2, 238.8 ± 19, and 390.1 ± 26 mW m^–2^ for the control, SA, and benzene-fed MFC, respectively.

Since there is no biofilm and no electron flow, the power density curve under abiotic conditions represents only the capacitive current. This is also evidenced by the absence of voltage generation in the MFC, as shown in Figure 2. However, when we introduced SA into the MFC, the power density increased by 49.8 times. The subsequent introduction of benzene as a substrate almost doubled (1.6 times) the power density compared to SA and provided a power density 80.6 times higher compared to abiotic conditions.

The power curve profiles reinforced those previously observed by cyclic voltammetry measurements (Figure S3), in which the hypothesis of biofilm formation plays an important role in the electron flow and energy generation in the system. However, it is worth highlighting that biofilm thickness and density can affect and interfere with electronic transfer, thereby offering greater resistance to electron flow, reducing diffusion, and limiting the increase in current.^69^

The internal resistance (*R*int) of the MFC is a crucial parameter and cannot be ignored or neglected.^69,72^ The *R*int values of each MFC were obtained by considering only the region corresponding to the ohmic drops (second region). *R*int values of 643 ± 58, 205 ± 42, and 146 ± 34 Ω were obtained for the control, SA-fed, and benzene-fed MFCs, respectively. The biofilm significantly decreased the Rint of the MFC by 3.1-fold for the SA-fed MFC and by 4.5-fold for the benzene-fed MFC, demonstrating once again that the biofilm formed was highly conductive and facilitated electron transfer.

In the absence of biofilm, the residual charge was 419.7 ± 157 mC. The charge increased substantially in the presence of biofilm to 6406.2 ± 341 and 9169.6 ± 493 mC for the SA-fed (Figure 4B – red line) and benzene-fed (Figure 4B – gray line) MFC, respectively. Compared to the control MFC (abiotic system), the residual charge increased 15.2- and 21.9-fold for the SA-fed and benzene-fed MFC, respectively.

Electrochemical impedance spectroscopy (EIS) highlights the anode surface changes before and after biofilm formation. EIS measurements were performed at OCP, and the potential values were 0.094, −0.461, and −0.460 V for the control, the MFC fed with SA, and benzene, respectively.

The EIS complex plane graphs (Figure S4) showed a semicircle at high frequencies, related to the charge transfer reaction, while the line at low frequencies was associated with the diffusion-limited oxidation process.^73^ For the control MFC, this line was not pronounced, indicating that diffusion was hampered. Moreover, the control MFC (Figure S4 −inset EIS spectrum – black) displayed a larger diameter semicircle and consequently a larger *R*ct (1442.8 Ω), suggesting that the carbon fiber brush (anode) surface hindered electron transfer.

The EIS complex plane for the MFC in the presence of biofilm (Figure S4) showed that the diameter of the semicircle decreased compared to the control MFC, resulting in *R*ct values of 28.5 and 9.6 Ω for the SA- and benzene-fed MFCs, respectively. The formation of a highly electroactive and conductive biofilm ultimately facilitates charge transfer. These *R*ct values confirmed that benzene promoted a more active and conductive biofilm, corroborating previous results.

Nevertheless, solution resistance (*R*s) decreased in the presence of biofilm, from 7.7 Ω (control MFC) to 1.25 Ω (SA-fed MFC) and 1.16 Ω (benzene-fed MFC), indicating that the biofilm made the solution less resistive. In addition, the double-layer capacitance (*Cdl*) increased slightly, from 0.11 mF cm^–2^ in the control MFC to 0.16 and 0.18 mF cm^–2^ in the SA- and benzene-fed MFC, respectively. *Cdl* increased in the presence of biofilm probably because the microorganisms formed a structured and dense nanowire network together with the carbon fiber brush bristles (Figure 1C,D). In the double layer, charge is formed by intra and extracellular electron storage mechanisms originating from the microorganism extracellular components, such as metabolites and/or physical appendages or pili. The resulting nanowire network would conduct the electrons, functioning as a nanonetwork with conductivity close to metallic conductivity and transferring electrons further away through the biofilm.^16^ ECSA values of 25.6, 37.2, and 41.9 cm^2^ was obtained for the carbon fiber brush without biofilm and in the presence of SA and benzene, respectively.

The stability and voltage, under maximum current flow (*R*ext = 0), of the MFCs fed with SA and benzene were evaluated. For this purpose, the anode and cathode potentials were measured using an Arduino, with an Ag/AgCl_(sat)_ (3.0 mol L^–1^ KCl) reference electrode, for 166 h. The cell voltage was obtained using eq 5:^16^

5 Ecell=Ecathode−Eanode

The results are shown in Figures S5 and S6 for the SA- and benzene-fed MFC, respectively. For both the cathode and anode, the potential was stable, with the voltage varying little during the period.

For the SA-fed MFC, the cathode and anode had average voltages of 801 mV and −282 mV vs Ag/AgCl_(sat)_ (3.0 mol L^–1^ KCl), respectively. For the benzene-fed MFC, the cathode and anode had average voltages of 860 mV and −289 mV vs. Ag/AgCl_(sat)_ (3.0 mol L^–1^ KCl), respectively. In the SA-fed MFC, the voltage remained practically constant throughout the monitoring period. The calculated cell voltage (519 mV) was close to the experimental cell voltage (549 mV), showing that the experimental and calculated values were in agreement. In the benzene-fed MFC, the voltage dropped slightly during the first 24 h because the biofilm was regenerated. Thereafter, the voltage increased significantly, with the maximum electron flow remaining practically constant. Furthermore, the calculated cell voltage (584 mV) was close to the experimental cell voltage (646 mV), showing that the experimental and calculated values were in agreement.

Finally, a comparison of the results obtained regarding the efficiency of benzene biodegradation and energy generation of the MFC proposed in this study was made with other devices in the literature. Table 1 compiles some recent MFC data on benzene treatment.

**Table 1:** Comparison of Data Obtained by Different MFCs during Benzene Biodegradation

Substrate (mg L^–1^)	Power Density (mW m^–2^)	Current Density (mA m^–2^)	Coulombic Efficiency (%)	Remove (%)	Ref.
Benzene (15.0) and ammonium (20.0)	316	990	14.0	100.0	^21^
Benzene (50.0)	38	100	-	97.4	^20^
Benzene (10.9)	0.021–0.449	0.66–20.44	0.22–7.59	100.0	^22^
Benzene (25.0) and acetate (1000.0)	320	2350	0.9	100.0	^14^
Benzene (800.0) and toluene (800.0)	0.67	60	0.11	97.0	^13^
Benzene (330.0)	390.1	2122.7	14.4	98.7	This study

The power density achieved by the MFC developed in this study was approximately 100 times greater than that reported for similar devices in the literature. Furthermore, it is noteworthy that the MFC generated a substantial current density (2122.7 mA m^–2^), indicating that the biofilm formed in this study was capable of producing significantly more energy when using both SA and benzene as substrates, while simultaneously facilitating the removal of these compounds. This finding represents a significant advancement in the development of technologies for the treatment of water contaminated with benzene.

### 3.3 Microbial Community in the Mangrove Sediment and MFC Bioanode

The microbiota composition and diversity indices were analyzed under three distinct conditions, including the mangrove sediment (inoculum) and the biofilms acclimated with either SA or benzene. These analyses provided insights into the microbial communities formed under different substrate conditions and their relationship with energy generation. The results are illustrated in Figure 5.

**Figure 5:**
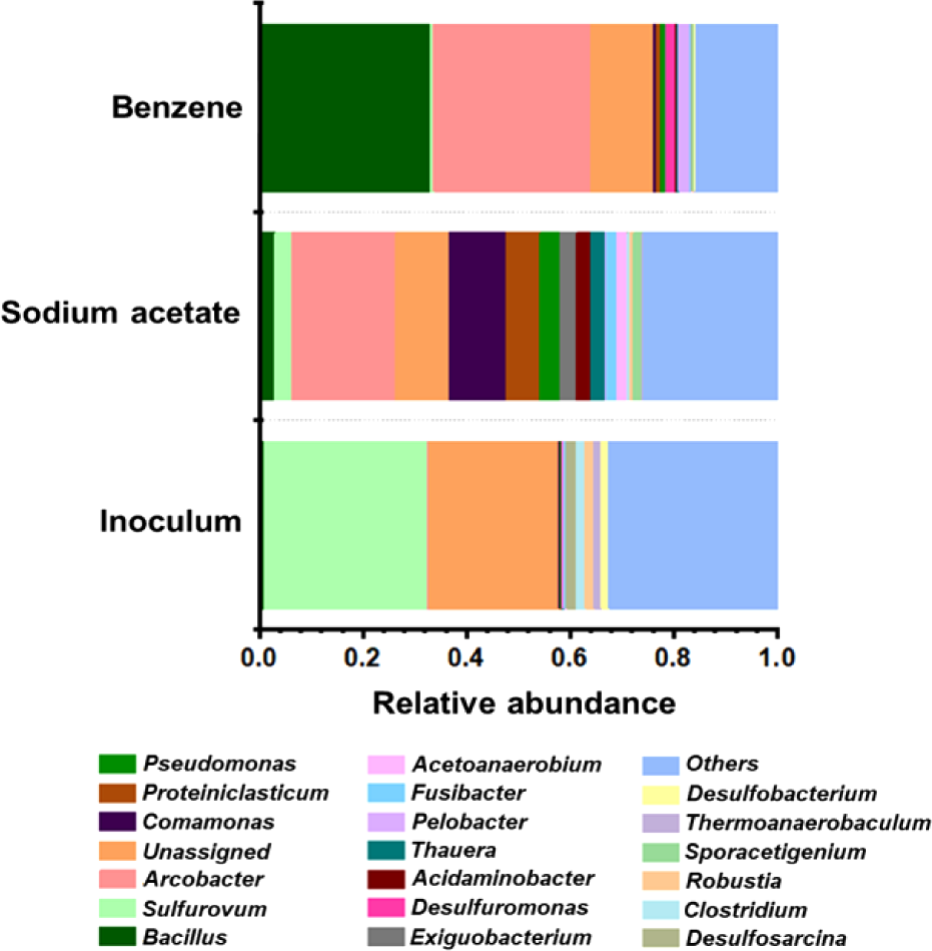
Relative abundance of various taxa at the genus level in the mangrove sediment and in the biofilms formed in MFCs fed with SA and benzene.

Mangrove microbial communities include all types of microorganisms, such as algae, fungi, and bacteria. However, sulfate-reducing bacteria stand out because they form an important group in anaerobic sediments.^74^ In the inoculum from mangrove sediment, the genus *Sulfuvorum* (31.45%) predominated, but a significant portion of the microorganisms remained unassigned (25.17%). Besides *Sulfuvorum*, other sulfur-reducing bacteria were detected in inoculum, inclusing *Desulfosarcina* (1.88%), *Clostridium* (1.76%), *Romboutsia* (1.69%), and *Desulfobacterium* (1.38%), which was expected for a mangrove sediment. *Sulfuvorum* is a facultative anaerobic and mesophilic genus that can oxidize hydrogen and reduce sulfur, nitrate, and thiosulfate.^75,76^ The mangrove environment may explain the high abundance of this genus: its high salinity and low oxygenation favor intermediates of anaerobic organic matter degradation, such as hydrogen and sulfides.^74^

The genus *Sulfuvorum*, the most abundant genus in the mangrove sediment (inoculum), became less abundant in the SA- and benzene-fed MFC biofilms (3.15% and 0.68%, respectively), which can be explained by the low sulfate content in the culture medium. In the SA-fed MFC biofilm, the predominant genera were *Arcobacter* (20.23%) and *Comamonas* (11.03%). A significant portion of the microorganisms remained unassigned (10.26%), and the genera *Proteinclasticum*, *Pseudomonas*, and *Desulfuromonas* were present at 6.53, 3.88, and 3.18%, respectively (Figure 5). Thus, genera *Arcobacter* and *Comamonas* predominated in the biofilm formed in the MFC supplemented with the preferred substrate of electrogenic microorganisms. The genus *Arcobacter* relative abundance increased from 0.44% in the inoculum to 20.23% in the SA-fed MFC biofilm.

*Arcobacter* tolerates small amounts of oxygen and has been reported to be the dominant genus in some MFCs.^55,77^ On the basis of the increased abundance of a flagellin protein under anaerobic growth on an insoluble electrode, *Arcobacter* itself can transfer electrons to an external solid electron acceptor.^78,79^ Additionally, some *Arcobacter* species reduce Mn and Fe,^78,79^ which indicates that they may be electrogenic, so the genus *Arcobacter* certainly participated in energy generation in the MFC. The *Comamonas* relative abundance increased from 0.1% in the inoculum to 11.03% in the SA-fed MFC biofilm, suggesting that the presence of this genus probably depended on SA. Indeed, Lim et al.^80^ correlated the genera present in MFC biofilms with exoelectrogenic activity and identified seven predominant genera, including *Comamonas*.

In the benzene-fed MFC biofilm, *Bacillus* (32.93%) and *Arcobacter* (30.28%) predominated, comprising 63.21% of the microbial community. The genera *Pelobacter*, *Desulfurumonas*, and *Pseudomonas* were also identified at 2.22, 1.64, and 0.87%, respectively (Figure 5). Clearly, benzene addition has shifted the microbial community composition by diminishing *Commamonas* and enriching the *Arcobacter* and *Bacillus* populations. This bacterial consortium also shaped the MFC performance, as confirmed in the electrochemical tests and characterizations (Section 3.2). For example, the electrogenic *Arcobacter* abundance increased from 20.23% to 30.28%, and the MFC power density increased from 238.8 ± 19 to 390.1 ± 26 mW m^–2^ in the SA- and benzene-fed MFC biofilm, respectively.

The most expressive enrichment is observed in the genus *Bacillus* which relative abundance increases from 0.88 to 3.0% and then to 32.93% in the inoculum, SA-fed MFC biofilm, and benzene-fed MFC biofilm, respectively. The genus *Bacilllus* is therefore important for degrading benzene.

The Shannon diversity index is the most widespread measure of diversity. The higher the Shannon index, the greater the diversity. The biofilm acclimated with SA (Figure S7) showed greater diversity, with an index of 5.73, followed by the mangrove sediment, with an index of 5.12, and the biofilm acclimated with benzene, with an index of 4.97. The lowest value of the Shannon index obtained for benzene was expected because the toxicity of this compound severely selects species. Similar results were observed when calculating the Simpson index (Figure S8), which is another important indicator that deals with species equitability and whose values are inversely related to biodiversity.^81^

In general, river and marine sediments present a great diversity of exoelectrogenic microorganisms because they contain a large amount of organic matter and offer favorable anaerobic conditions.^82^ However, numerous studies have focused on *Shewanella* sp. and *Geobacter* sp., providing limited information about the molecules, structures, extracellular electron transfer capacity, and electron transfer mechanisms of exoelectrogenic microorganisms.^82^ Given that the mangrove sediment sample comes from a region in the Southern Hemisphere, its overall composition differs greatly from anything previously described in the literature. For example, species belonging to the genus *Pseudomonas* sp. are reported to be promising microorganisms for degrading aromatic compounds and various substrates.^83^ However, in our analysis, the genus *Pseudomonas* corresponded to only 3.88% and 0.87% of the microbial community in the SA- and benzene-fed MFC, respectively. Therefore, the changes in the microbial community observed when the substrate was changed suggested that different metabolic pathways were activated within the same community, which allowed the MFCs to exhibit distinct electrochemical behavior.

Over the past decade, studies have demonstrated that strains capable of degrading benzene, toluene, ethylbenzene, and xylenes (BTEXs) in the environment belong to the genus *Pseudomonas*, for example, *Pseudomonas putida* and *Pseudomonas fluorescens*.^30,31^ On the other hand, Wongbunmak et al.^28^ reported a Gram-positive bacterial isolate identified as *Bacillus amyloliquefaciens* subsp. plantarum strain W1 that can degrade all BTEXs. The authors also proposed the degradation pathway of these compounds by the new strain: benzene was converted to benzene dihydrodiol by dioxidation and to phenol by monooxidation. The identified degradation products were pyruvate or acetaldehyde, which were subsequently incorporated into the TCA cycle. It is worth noting that the catabolic pathways for BTEX biodegradation have been well characterized in Gram-negative bacteria but not in Gram-positive bacteria, especially in the genus *Bacillus*.

Considering that the genus *Bacillus* predominated in the MFC biofilm fed with benzene, the phylogenetic diversity of this genus was analyzed (Figure S9). To date, there are no references on *Bacillus* in an MFC biofilm that specifically degrades benzene and produces bioelectricity.

### 3.4 Monitoring Benzene Biodegradation

Figure 6 shows the benzene biodegradation and cell voltage (A) and the concentration of the products formed from benzene biodegradation as a function of time (B).

**Figure 6:**
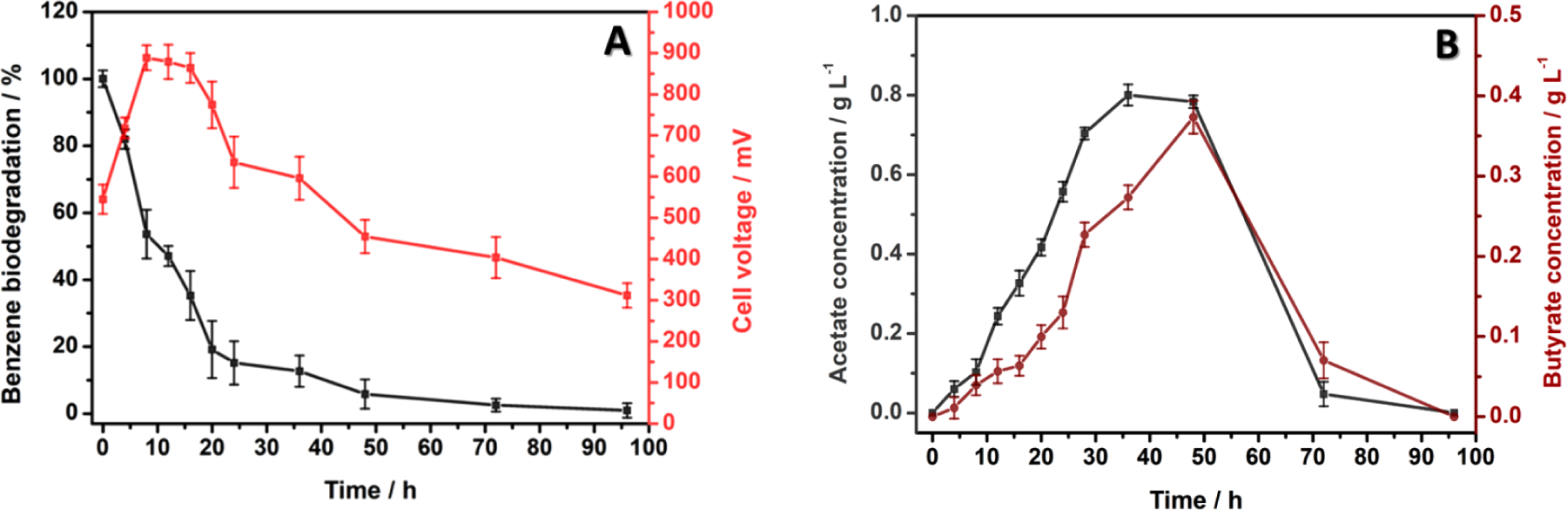
Benzene biodegradation and cell voltage (A) and concentration of the products formed from benzene biodegradation as a function of time (B).

According to Wongbunmak et al.,^28^ *B. amyloliquefaciens* W1 degrades benzene through two main routes: dioxidation to benzene dihydrodiol or monooxidation to phenol. In this process, the authors identified enzymes such as benzene 1,2-dioxygenase, toluene dioxygenase, and naphthalene dioxygenase, as well as reaction intermediates such as benzene dihydrodiol, phenol, catechol, 2-hydroxymuconic semialdehyde, *cis*-muconate, 1,2,3-trihydroxybenzene, *cis*-2-hydroxypenta-2,4-dienoate, and 4-hydroxy-2-oxovalerate.

Benzene biodegradation was monitored in the MFCs by GC-FID. The chromatograms (Figure S10) showed that the benzene peak (*t*_R_ = 1.32 min) gradually decreased, confirming its biodegradation throughout the MFC operation (96 h).

The HPLC-RID chromatogram obtained for the products (Figure S11) revealed the presence of two compounds: acetate (*t*_R_ = 15.3 min) and butyrate (*t*_R_ = 21.7 min). The acetate and butyrate concentrations increased gradually until 48 h. Thereafter, these products were also consumed, and their peaks disappeared after 96 h.

The products present in the gaseous fraction were monitored by GC-TCD after treatment for 96 h. The chromatogram (Figure S12) showed four peaks, corresponding to hydrogen (*t*_R_ = 0.90 min), nitrogen (*t*_R_ = 1.76 min) (originating from the MFC deaeration process), methane (*t*_R_ = 4.45 min), and carbon dioxide (*t*_R_ = 11.99 min).

The percentage of benzene in the MFC decreased over time to 98.7 ± 2.4% after 96 h. Therefore, the microorganisms in the MFC biofilm biodegraded benzene while generating energy. The observed drop in product concentration may have been due to its reassimilation by microorganisms present in the MFC.

The CE calculated for MFC was 14.4%. However, the efficiency of complex and interconnected processes, such as benzene degradation in MFC, can also be assessed with the help of the concept of exergy through the so-called exergy analysis. Exergy analysis is a thermodynamic tool that allows the assessment of useful effects, resource destruction, and losses to the environment based on a common basis: the amount of work available. Conceptually, exergy can be defined as the amount of work obtained when a fragment of matter is brought to a state of thermodynamic equilibrium with the common components of its surroundings through reversible processes.^84^

An instantaneous performance metric for the operational efficiency of the MFC can be defined based on the electrical power supplied by the fuel cell and the exergy input corresponding to the consumption of benzene at the anode:^84^

6 $$\eta =\frac{\delta W_{e}}{\frac{\partial Ex}{\partial N_{b}}.\frac{dN_{b}}{dt}}$$

where *W*_*e*_ corresponds to the work delivered by the fuel cell, *Ex* to the total exergy of the anode electrolyte, and *N*_b_ to the number of benzene moles within the anode electrolyte.

One should observe that the instantaneous exergy efficiency is governed by two main phenomena with a time delay between them: the denominator, representing the consumption of the benzene by the biofilm, and the numerator, which represents the subsequent energy release by the biofilm associated with the electric power yield. Under these circumstances, the use of a cumulative efficiency would provide better insight into the MFC operational efficiency:^84^

7 $$\eta =\frac{\int_{t=0}^{t=t_{cycle}}\delta W_{e}dt}{\int_{t=0}^{t=t_{cycle}}\frac{\partial Ex}{\partial N_{b}}.\frac{dN_{b}}{dt}}=\frac{W_{e}}{Ex_{b}}$$

where W*_e_* represents the electrical work produced by the MFC during one feed cycle, while Ex*_b_* corresponds to the exergy received by the biofilm through benzene biodegradation within this same cycle.

Figure 7 shows the results obtained for the benzene exergy and electrical energy released by the cell (A) and the cumulative energy efficiency of the MFC (B).

**Figure 7:**
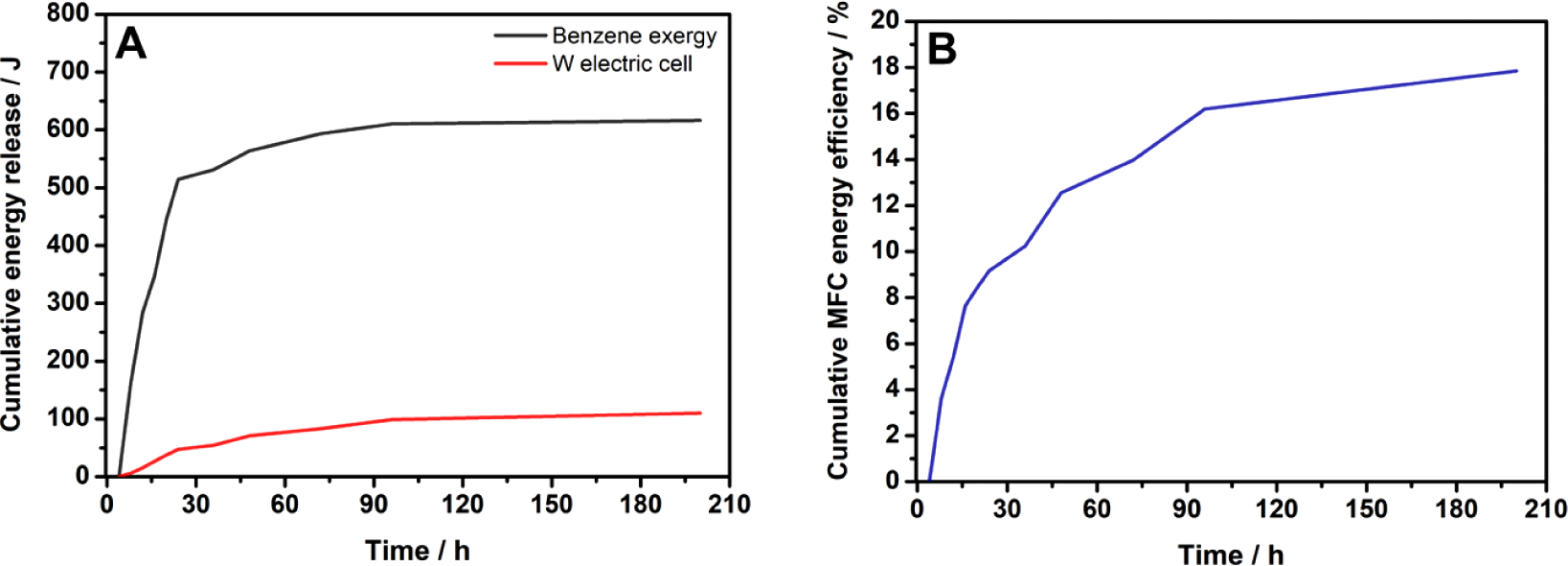
Benzene exergy and electrical energy released by the cell (A) and cumulative MFC energy efficiency (B).

The red curve in Figure 7A indicates that the exergy value obtained from the benzene consumed by the microorganisms present in the biofilm, over 196 h of treatment, reached a maximum limit of 616.1 J. This value represents the equivalent, in terms of work (W), of the energy contained in the “fuel” (benzene) consumed by the biofilm. In contrast, the black curve shows the electrical energy released by the microbial fuel cell (MFC), which reached a maximum value of 109.9 J.

By dividing the exergy value of the benzene by the value of the electrical energy generated by the MFC, the MFC cumulative energy efficiency was calculated, amounting to 17.8% over 196 h of operation. This result is quite close to the value found for the Coulombic efficiency of the MFC (14.4%), confirming that both Coulombic efficiency and exergy analysis are reliable metrics for expressing MFC performance.

However, this cumulative energy efficiency of 17.8% indicates that approximately one-fifth of the total potential for electrical power generation was effectively utilized by the MFC. This means that part of the chemical energy made available by the MFC, through substrate consumption, was directed toward cell growth.^29^ Therefore, the voltage generated by the MFC (Figure 3A) appears to originate mainly from the faradaic process associated with benzene consumption and biodegradation promoted by the microorganisms in the biofilm.

Furthermore, the Coulombic efficiencies (CEs) obtained for MFCs using benzene alone or in combination with other substrates varied significantly between studies (Table 1). The highest CE value obtained was for the system proposed in this study (14.4%), surpassing the other configurations and highlighting the potential of the system for enhanced energy recovery from highly toxic pollutants.

### 3.5 Acute Toxicity Tests

Initially, tests on pure Lovley and Phillips medium were performed to verify whether the medium was toxic to *D. similis*. The results (Table S1) showed high toxicity of the medium, caused by the large amount of salts present.

Regarding the tests performed with diluted Lovley and Phillips medium, the highest dilution that did not show immobility was 3.3% (v/v) (Table S2). Therefore, this concentration was chosen to evaluate the toxicity of the medium in the presence of benzene and after treatment in MFC.

Subsequently, toxicity in the presence of benzene at 0.33, 1.1, 3.3, 11.0, and 33.0 mg L^–1^ was evaluated. The results obtained (Table S3) showed that benzene at 11.0 and 33.0 mg L^–1^ presented acute toxicity to *D. similis*, with average mortality rates of 95.0 and 100.0%, respectively. Finally, toxicity after treatment in MFC was evaluated. The results obtained (Table S4) showed that the treatment completely removed toxicity and did not generate any toxic metabolites for *D. similis* under the conditions tested.

## 4 Conclusion

In this study, an MFC system was developed using a novel electrogenic microbial consortium derived from mangrove sediments for the bioremediation of benzene and the simultaneous generation of energy. The biofilm formed, composed mainly of the genera *Bacillus* (32.9%) and *Arcobacter* (30.3%), exhibited remarkable adaptation to benzene as a substrate, promoting high conductivity and electrochemical stability. As a result, the MFC achieved 98.7 ± 2.4% benzene removal within 96 h, a maximum voltage of 902.3 ± 20.6 mV, a power density of 390.1 ± 26 mW m^–2^, and a cumulative energy efficiency of 17.8% over 196 h of operation—values significantly higher than those reported for similar systems. Furthermore, toxicity tests demonstrated the complete removal of solution toxicity after treatment.

Despite these promising results, the study has limitations inherent to the laboratory scale and batch mode. Future research will focus on the implementation of continuous and larger-scale reactors, the evaluation of system performance in real effluents and complex contaminant mixtures, and the development of strategies to increase energy efficiency, including electrode engineering.

In summary, the results demonstrate that microbial consortia derived from mangroves represent a highly promising strategy to integrate efficient bioremediation of aromatic compounds with bioelectricity generation, contributing to the advancement of bioelectrochemical technologies applied to environmental decontamination.

## Supplementary Material



## Notes

AdThe Supporting Information is available free of charge at https://pubs.acs.org/doi/10.1021/acsomega.5c05703.

Additional results including graphs, cyclic voltammograms, electrochemical impedance spectra, chromatograms, phylogenic tree, and tables (PDF)
