# Supplementary material for: High-Performance Microbial Fuel Cell for Aromatic Hydrocarbon Bioremediation: Leveraging a Unique Mangrove-Derived Electrogenic Consortium
Source: ACS Omega. 2025 Oct 22;10(43):51074–87. doi: 10.1021/acsomega.5c05703 (PMC12593025; doi:10.1021/acsomega.5c05703)
Supplement: Supplementary file 1 [file ao5c05703_si_001.pdf]

Supplementary material

HIGH-PERFORMANCE MICROBIAL FUEL CELL FOR AROMATIC HYDROCARBON BIOREMEDIATION: LEVERAGING A UNIQUE MANGROVE-DERIVED ELECTROGENIC CONSORTIUM

João Carlos de Souza[a], Ana Clara Bonizol Zani[a], João Pedro Silva[b], Amanda dos Santos[c], Gisela de Aragão Umbuzeiro[c], André Valente Bueno[d], Fernanda Leite Lobo[d], Valeria Reginatto[a] and Adalgisa Rodrigues de Andrade[a*]

[a]University of São Paulo (USP), Faculty of Philosophy, Sciences and Letters at Ribeirão Preto (FFCLRP), Department of Chemistry

Avenida Bandeirantes, 3900, Ribeirão Preto - 14040-900, São Paulo State, Brazil

[b]São Paulo State University (UNESP), Institute of Chemistry, Department of Analytical, Physical-Chemical and Inorganic Chemistry

Rua Prof. Francisco Degni, 55, Araraquara - 14800-060, São Paulo State, Brazil

[c]State University of Campinas (UNICAMP), Faculty of Technology

Rua Paschoal Marmo, 1888, Limeira – 13484-332, São Paulo State, Brazil

[d]Federal University of Ceará, Technology Center, Department of Mechanical Engineering

Avenida da Universidade, 2853, Fortaleza - 60020-181, Ceará State, Brazil

[*]Corresponding Author

Address: Avenida Bandeirantes, 3900, Ribeirão Preto - 14040-900, São Paulo State, Brazil

E-mail: ardandra@usp.br

Phone: + 55 16 3315-3725

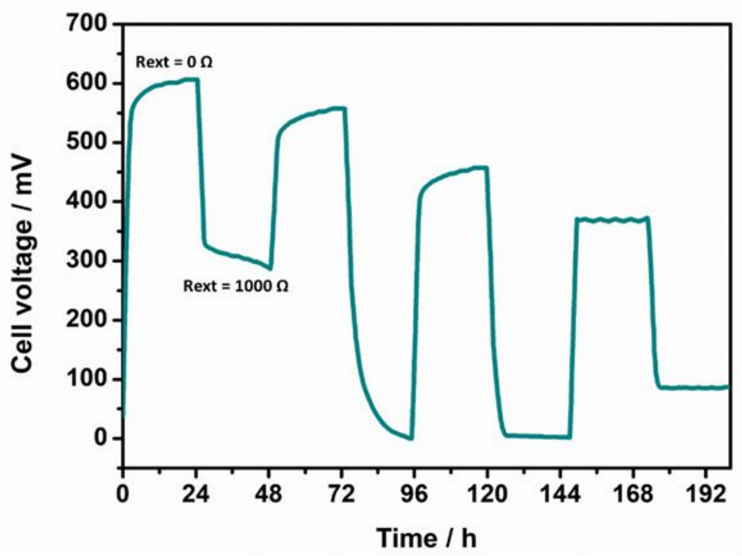

Figure S1. MFC voltage measurement by alternating external resistance, with 24 h intervals, in the presence of 1000.0 mg L^{-1} SA. Conditions: (Rext = 1000 Ω and Rext = 0).

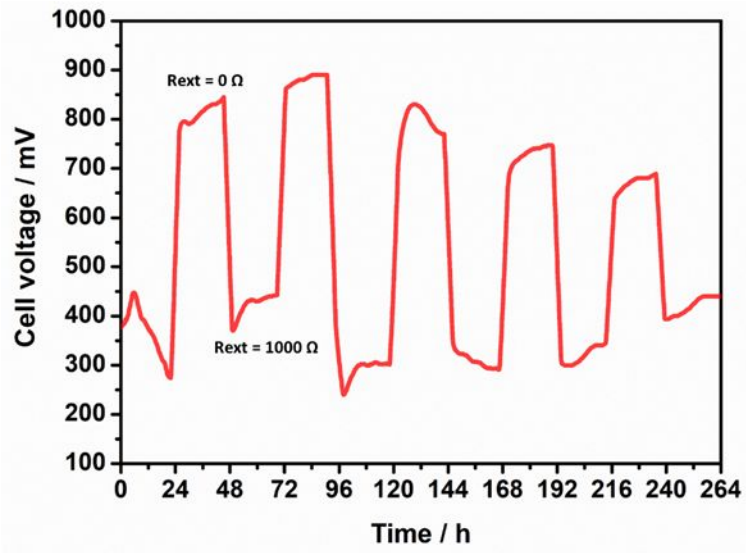

Figure S2. MFC voltage measurement by alternating external resistance, with 24 h intervals, in the presence of 330.0 mg L^{-1} benzene. Conditions: (Rext = 1000 Ω and Rext = 0).

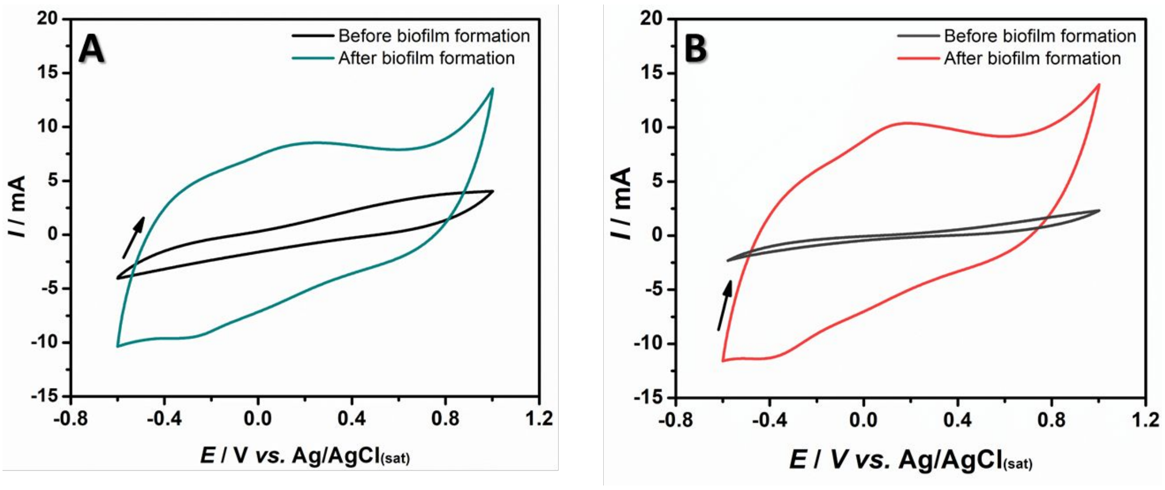

Figure S3. Cyclic voltammograms obtained for the anode, before and after biofilme formation, in Lovely and Phillips culture medium with 1000.0 mg L^{-1} SA (A) and 330.0 mg L^{-1} benzene (B). Scan rate (ν) = 1 mV s^{-1}.

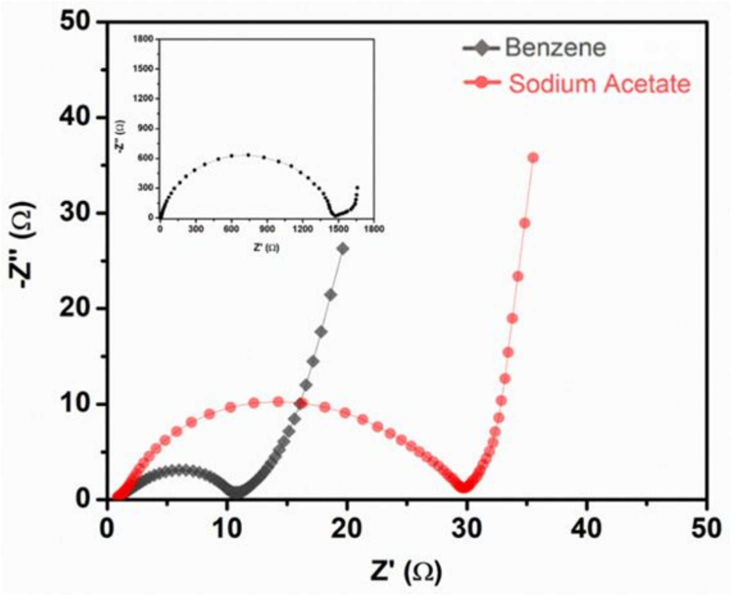

Figure S4. EIS spectra obtained for the control MFC (black - insert), SA fed MFC (red), and benzene fed MFC (gray) in support electrolyte composed of Lovley and Phillips culture medium.

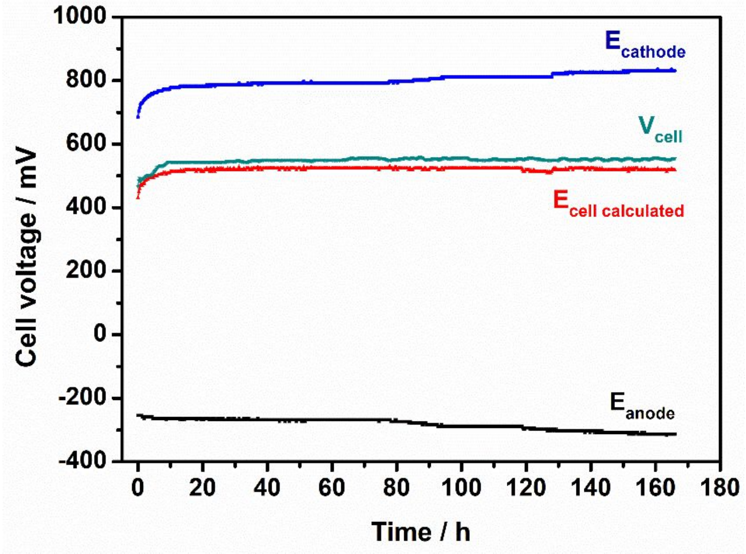

Figure S5. Assessment of the anodic, cathodic, MFC calculated, and MFC experimental potentials as a function of time. Conditions: 1000.0 mg L^{-1} SA; Rext = 0. $E_{cell\ calculated}$ = $E_{cathode}$ – $E_{anode.}$

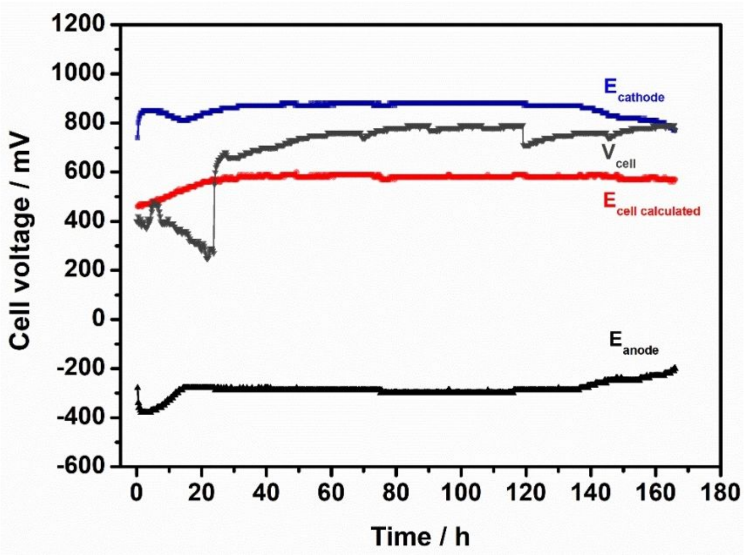

Figure S6. Assessment of the anodic, cathodic, MFC calculated, and MFC experimental potentials as a function of time. Conditions: 330.0 mg L^{-1} benzene; Rext = 0. $E_{cell\ calculated} = E_{cathode} - E_{anode}$.

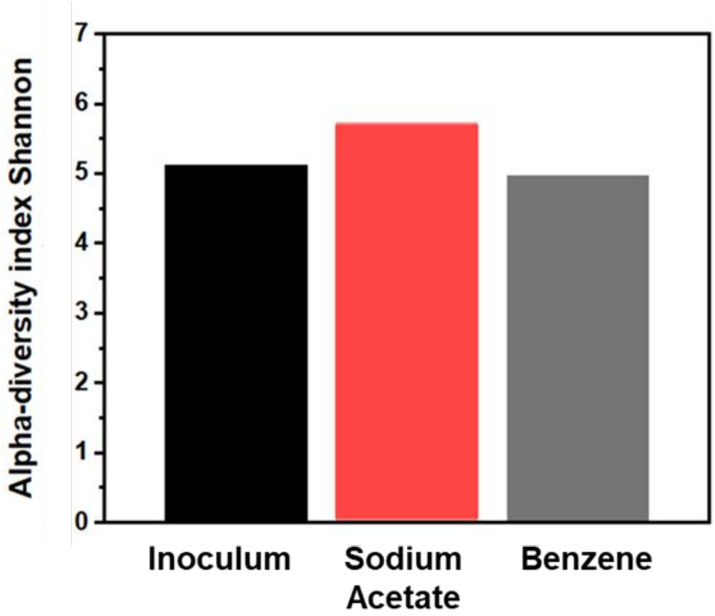

Figure S7. Alpha-diversity in the inoculum, SA-fed MFC biofilm, and benzene-fed MFC biofilm calculated by using the Shannon index.

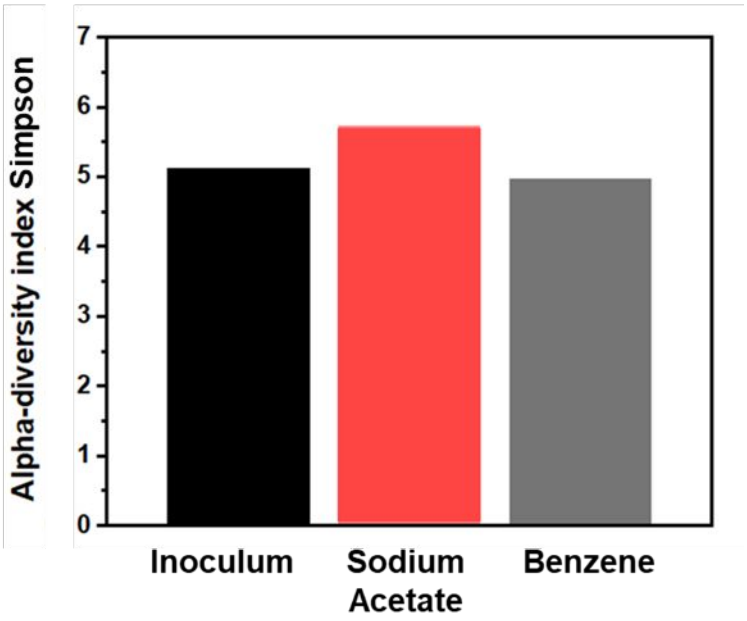

Figure S8. Alpha-diversity in the inoculum, SA-fed MFC biofilm, and benzene-fed MFC biofilm calculated by using the Simpson index.

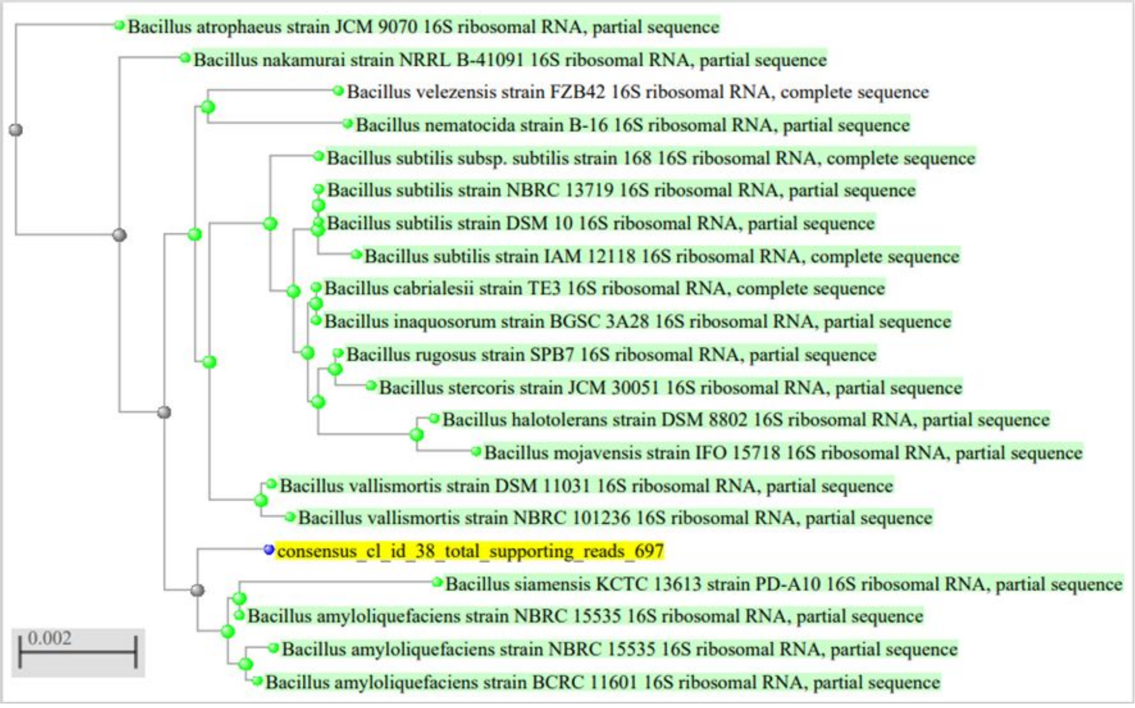

Figure S9. Phylogenic tree of *Bacillus* species based on comparative analysis of the 16S rRNA sequences recovered from the benzene fed MFC biofilm.

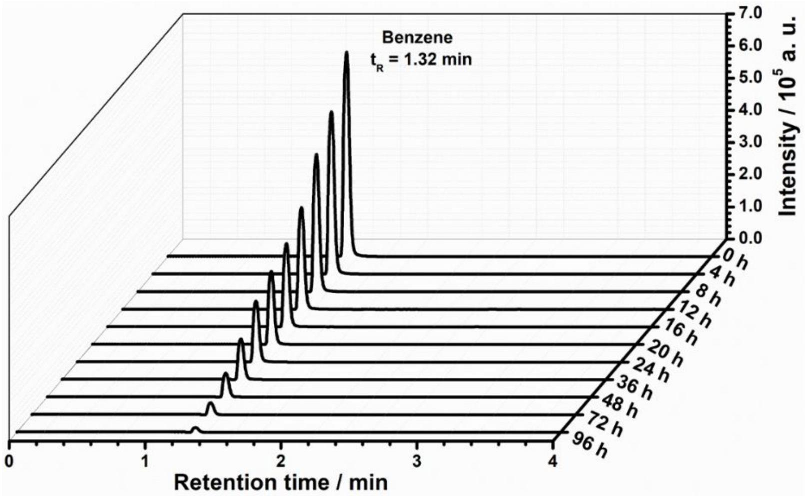

Figure S10. GC-FID chromatograms obtained for benzene biodegradation in the MFC over 96 h. Conditions: carrier gas = nitrogen (99.995% purity), flow rate = 1.0 mL min^{-1}, Restek Rtx chromatographic column – Biodiesel TG (Crosslinked 5% diphenyl – 95% dimethyl polysiloxane; 10 m length × 0.32 mm i.d., 0.10 μm df), column temperature = 40.0 °C, split mode (1:50), and detector temperature = 240.0 °C.

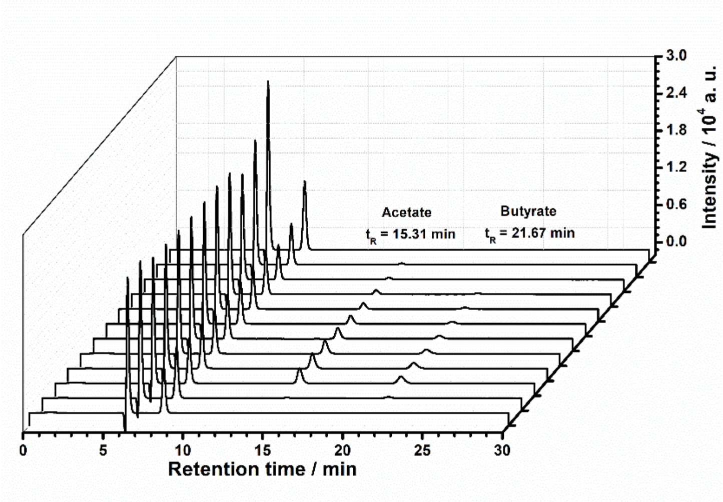

Figure S11. HPLC-RID chromatograms obtained for the products in solution formed during benzene biodegradation in the MFC over 96 h. Conditions: mobile phase H_2SO_4 5.0 mmol L^{-1}, Aminex HPX-87H ion exclusion column (300 × 7.8 mm), flow rate 0.6 mL min^{-1}, temperature of column 60.0 °C, and volume injection of 10.0 μL.

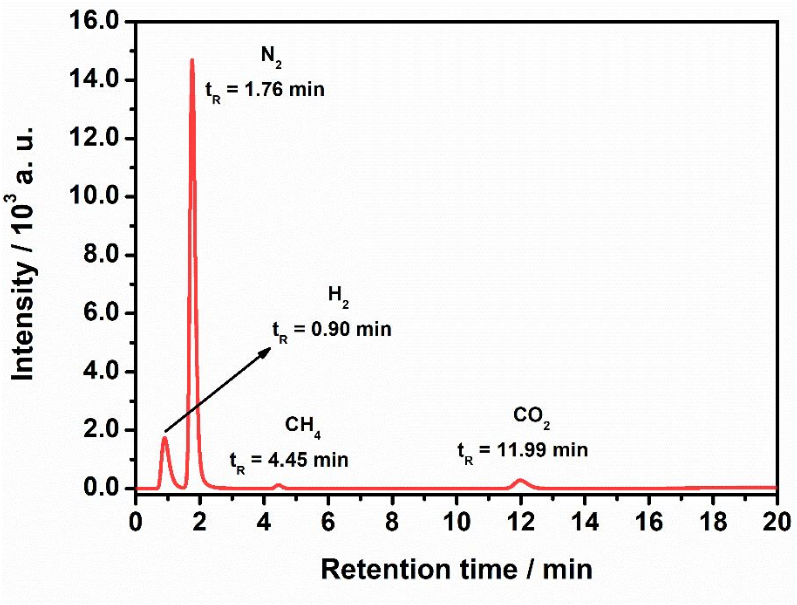

Figure S12. GC-TCD chromatogram obtained for the gaseous fraction of benzene biodegradation products in MFC. Conditions: carrier gas = argon (99.995% purity), flow rate = 10.0 mL min^{-1}, Restek Shin Carbon ST– Micropacked chromatographic column (SilcoSmooth Tubing Mesh: 100/120 OD: 1/16"; 2 m length × 1.0 mm i.d.), column temperature = 50.0 °C, splitless mode, detector temperature = 80.0 °C and injection volume of 50.0 µL.

Table S1. Acute toxicity testing against *Daphnia similis* of pure Lovley and Phillips medium (without benzene) and after degradation in the MFC (48 h).

**Sample**	**N. of immobile organisms**	**Immobility (%)**	**Parameters**			
			**pH (a. u.)**		**Conductivity**	
			**Initial**	**Final**	**Initial**	**Final**
Control	0/20	0	6.59	6.83	250.2 µS cm^{-1}	249.8 µS/cm^{-1}
Lovley and Phillips medium	20/20	100	8.89	9.00	5.89 mS cm^{-1}	5.80 mS cm^{-1}

*n = 2

Table S2. Acute toxicity test against *Daphnia similis* of diluted Lovley and Phillips medium and after benzene degradation in the MFC (48 h).

Concentration (%)	N. of immobile organisms	Immobility (%)	Parameters			
			pH		Conductivity (µS cm^{-1})	
			Initial	Final	Initial	Final
Control	0/20	0	7.69	6.53	252.6	255.8
0.1	0/20	0	7.47	6.79	258.3	265.1
0.3	0/20	0	7.81	7.68	278.5	278.6
1.0	0/20	0	8.06	7.11	319.0	319.0
3.3	2/20	10	8.44	8.16	480.0	491.0
10	20/20	100	8.74	8.51	864.0	877.0

*n = 2

Table S3. Acute toxicity test of Lovley and Phillips medium with benzene, using *Daphnia similis* (48h).

Benzene concentration (mg L^{-1})	N. of immobile organisms	Immobility (%)	Parameters			
			pH		Conductivity (µS cm^{-1})	
			Initial	Final	Initial	Final
Solvent control[a]	0/20	0	6.60	6.46	256.9	257.7
0.3	0/20	0	6.58	6.54	270.1	269.2
1.0	0/20	0	8.11	7.26	319.0	323.0
3.3	0/20	0	7.63	7.23	386.0	386.0
11.0	19/20	95	7.86	7.95	554.1	558.3
33.0	20/20	100	8.31	8.34	869.0	855.0

[a] DMSO at 0.01%;
*n = 2

Table S4. Acute toxicity test against *D. similis* of the Lovley and Phillips medium after treatment to remove benzene (48h).

Sample	N. of immobile organisms	Immobility (%)	Parameters			
			pH		Conductivity (µS cm^{-1})	
			Initial	Final	Initial	Final
Control	1/20	5	6.60	6.56	256.9	257.4
Lovley and Phillips medium after MFC treatment	0/20	0	8.27	8.25	875.0	878.0

*n = 2
